# Machine learning models using serum gastric biomarkers for the non-invasive prediction of atrophic gastritis: a comparative study

**DOI:** 10.3389/fmed.2026.1757004

**Published:** 2026-02-18

**Authors:** Dong Li, Haitao Yu, Baihan Jin, Dongfang Dong, Lingxue Cheng, Wenzhu Dong

**Affiliations:** Department of Gastroenterology, No. 971 Hospital of the People’s Liberation Army Navy, Qingdao, Shandong, China

**Keywords:** AUC, Brier score, calibration, chronic atrophic gastritis, Decision Curve Analysis, machine learning, pepsinogen

## Abstract

**Background and aims:**

The early, non-invasive detection of chronic atrophic gastritis (CAG), a precancerous lesion, remains a clinical challenge. While serological biomarkers are promising alternatives to endoscopy for screening, their predictive accuracy using conventional methods is suboptimal. This study aimed to identify key predictors of CAG and to comparatively develop multiple machine learning (ML) models, evaluating whether ML offers a definitive advantage and identifying a reliable model for triaging patients to endoscopy.

**Methods:**

In this retrospective diagnostic study (conducted from January to October 2020), 222 subjects (CAG prevalence: 30.6%) were stratified randomly into a training set (80%) and an independent test set (20%). Feature selection was performed exclusively on the training set using multivariate logistic regression, which identified four independent predictors: PGI, the PGI/PGII ratio, age, and anti-*H. pylori* antibody status. Using these predictors, eight models—including Logistic Regression (as baseline), Elastic Net, Support Vector Machine, Neural Network, and tree-based ensembles—were trained and optimized via 5-fold cross-validation. Model performance was rigorously evaluated on the held-out test set using discrimination (AUC, sensitivity, specificity), calibration (Brier score), and clinical utility (Decision Curve Analysis).

**Results:**

Multivariable analysis identified the four predictors, with anti-*H. pylori* antibody positivity associated with an approximately four-fold higher odds of CAG. On the independent test set, the Elastic Net (AUC = 0.823) and Logistic Regression (AUC = 0.810) models demonstrated the highest and most robust discriminative performance, showing excellent sensitivity (0.923) and negative predictive value (>0.95) for ruling out CAG. Statistical comparison confirmed that their AUCs were significantly higher than those of the severely overfitted tree-based models (e.g., Random Forest), but not significantly different from other complex models like Support Vector Machine. Decision Curve Analysis confirmed the superior net clinical benefit of the Elastic Net and Logistic Regression models across a wide range of decision thresholds.

**Conclusion:**

Simple, interpretable linear models (Elastic Net and Logistic Regression) based on four routine clinical parameters provide a robust tool for the non-invasive identification of CAG in a clinical population referred for endoscopic evaluation. They show particular strength in ruling out disease, supporting their potential role as a triage tool. In this setting, they demonstrated more consistent performance than more complex machine learning algorithms. External validation in broader populations is warranted to confirm generalizability before clinical implementation.

## 1 Introduction

Chronic atrophic gastritis (CAG), characterized by the loss of gastric glands and/or intestinal metaplasia, is a well-established precancerous lesion for gastric cancer (GC) (1). The early detection and management of CAG are critical for interrupting the GC cascade, particularly in regions with a high GC burden. *Helicobacter pylori* (*H. pylori*) infection is the primary etiological driver, initiating chronic inflammation that, through molecular pathways such as NF-κB activation, progresses from CAG to malignancy (1, 2).

In clinical practice, the definitive diagnosis of CAG relies on histopathological examination of gastroscopic biopsies. However, the invasiveness, cost, and limited accessibility of endoscopy hinder its use for population-wide screening (3, 4). Consequently, non-invasive serological tests have been extensively investigated. Serum biomarkers, including pepsinogen I (PGI), pepsinogen II (PGII), the PGI/PGII ratio, gastrin-17 (G-17), and anti-*H. pylori* antibodies, form the basis of the “serological biopsy.” Notably, low PGI levels and a reduced PGI/PGII ratio correlate strongly with gastric corpus atrophy and GC risk, offering a promising tool for initial risk stratification (5–7).

Despite established associations, the predictive performance of these biomarkers, individually or in combination, for accurately identifying CAG remains suboptimal and inconsistently reported across studies (8–10). Most existing models rely on conventional statistical methods (e.g., logistic regression), which may not adequately capture complex, non-linear interactions among predictors. Machine learning (ML) algorithms are well-suited to model such intricate relationships and have shown promise in improving diagnostic accuracy (11, 12). Given the moderate sample size and structured nature of our dataset (*n* = 222 with five key biomarkers), we employed traditional ML models (e.g., SVM, Random Forests) rather than data-intensive deep learning, to ensure a better balance between model robustness, interpretability, and performance. A systematic comparison of diverse ML algorithms for CAG prediction using this specific biomarker panel is, however, lacking.

Therefore, this study aimed to: (1) identify independent serum and demographic predictors of CAG among PGI, PGII, the PGI/PGII ratio, G-17, anti-*H. pylori* antibody status, and age; and (2) construct, evaluate, and compare the performance of multiple ML models (including Support Vector Machine, Random Forest, XGBoost, and Neural Networks) against a traditional logistic regression baseline. Beyond merely developing a predictive model, we sought to critically assess whether increased algorithmic complexity inherently leads to better generalizable performance in this context, given a moderate sample size and a focused set of predictors. This comparative approach aims to provide empirical evidence to inform model selection for similar clinical prediction tasks. We sought to determine whether ML offers a definitive advantage for this task and to identify the most robust model for potential clinical application, such as triaging high-risk individuals for endoscopy in primary care settings, thereby optimizing resource use.

## 2 Methods

### 2.1 Study population and data source

This retrospective diagnostic study consecutively enrolled 222 subjects who underwent both gastroscopy and serum gastric function tests at the PLA Navy No. 971 Hospital between January and October 2020. Participants were categorized into an atrophic gastritis (AG) group or a non-AG control group based on the gold standard of gastric mucosal biopsy histopathology. The exclusion criteria were as follows: (1) previous *Helicobacter pylori* (*H. pylori*) eradication therapy; (2) history of gastric surgery; (3) use of proton pump inhibitors or H2 receptor antagonists within 2 weeks; (4) severe systemic diseases; (5) pregnancy; (6) history of malignancy; and (7) current use of antisecretory or anticoagulant medications. Fasting venous blood samples were collected from all participants in the morning prior to endoscopy. The study protocol was approved by the Institutional Ethics Committee of the PLA Navy No. 971 Hospital (Approval No.: 971LL-2019012).

### 2.2 Specimen processing and biomarker measurement

Serum was separated from fasting venous blood by centrifugation for the subsequent measurement of five gastric-specific circulating biomarkers: pepsinogen I (PGI), pepsinogen II (PGII), the PGI/PGII ratio, gastrin-17 (G-17), and anti-*H. pylori* IgG antibody. The serum levels of PGI, PGII, and G-17 were quantified using commercially available enzyme-linked immunosorbent assay (ELISA) kits (Pepsinogen I ELISA, Pepsinogen II ELISA, and Gastrin-17 ELISA, Snibe Diagnostic, Shenzhen, China). Anti-*H. pylori* IgG antibodies were qualitatively detected using a colloidal gold immunochromatographic assay (Anti-*H. pylori* kit, HUIAN, Shenzhen, China).

### 2.3 Gastroscopic examination and histopathological assessment

Gastroscopy with biopsy served as the gold standard for diagnosing AG. Following a standardized protocol, endoscopists obtained one biopsy sample each from the gastric antrum and gastric body along the greater curvature (10). Biopsy specimens were fixed in 10% neutral buffered formalin, embedded in paraffin, and sectioned routinely for hematoxylin–eosin (HE) staining and Alcian blue staining. The presence of *H. pylori* was assessed using modified Giemsa staining.

### 2.4 Data preprocessing and data splitting

The qualitative anti-*H. pylori* antibody results were converted into a binary variable (positive/negative) according to the manufacturer’s instructions. Continuous variables, including PGI, PGII, the PGI/PGII ratio, G-17, and age, were recorded as numerical values. The presence of chronic atrophic gastritis (CAG) was defined as the binary outcome (AG vs. non-AG). Records with any missing data for these variables were excluded from the analysis using complete-case analysis.

To ensure a fully independent evaluation and prevent data leakage, the entire cohort was first randomly split into a training set and an independent hold-out test set. The split was performed using the createDataPartition function from the caret package (version 6.0–94) in R, which implements stratified random sampling based on the CAG outcome variable. This ensured that the proportion of CAG cases was preserved in both sets. The split ratio was set to 80% for training and 20% for testing.

All subsequent steps of feature selection, model development, and hyperparameter tuning were conducted exclusively using the training set. The test set remained completely untouched until the final, single evaluation of the locked models.

Any data preprocessing (e.g., feature scaling) was fit solely on the training set, and the resulting parameters were then applied to the test set. For algorithms requiring feature scaling (e.g., logistic regression, support vector machine, elastic net, and neural network), features were standardized to z-scores based on the training set’s mean and standard deviation; tree-based models were trained on unscaled data.

### 2.5 Feature selection and model development (conducted on the training set)

#### 2.5.1 Descriptive statistics and univariate screening

Descriptive statistics and univariate analyses comparing CAG vs. non-CAG groups were performed on the training set. Continuous variables were tested for normality using the Shapiro–Wilk test. Normally distributed data are presented as mean ± standard deviation and compared using the independent samples t-test. Non-normally distributed data are presented as median (interquartile range) and compared using the Wilcoxon rank-sum test. Categorical variables are expressed as numbers (percentages) and compared using the Chi-square test or Fisher’s exact test, as appropriate. A *p*-value < 0.05 was considered statistically significant for this screening step.

#### 2.5.2 Predictor selection via multivariate logistic regression

Variables with *p* < 0.05 in the univariate analysis of the training set were entered into a multivariate logistic regression model (with CAG as the dependent variable) fitted only on the training data. Adjusted odds ratios (aORs) with 95% confidence intervals (CIs) were calculated, and multicollinearity was assessed using the variance inflation factor (VIF), with VIF > 10 indicating problematic multicollinearity. Based on statistical significance (*p* < 0.05) in this training-set model, the final predictor set was selected and locked for all subsequent modeling. This resulted in the inclusion of: PGI, the PGI/PGII ratio, age, and anti-*H. pylori* antibody status. PGII was excluded as it did not reach significance at this stage.

#### 2.5.3 Machine learning modeling

Using the locked set of predictors identified above, the following machine learning models were developed and tuned exclusively within the training set framework using 5-fold cross-validation:

Logistic Regression (LR): Served as the baseline model.

Elastic Net: The mixing parameter (*α*) was set to 0.5; the regularization strength (*λ*) was optimized via cross-validation.

Random Forest (RF): Configured with 1,000 trees and a minimum of 5 samples in terminal nodes.

Support Vector Machine (SVM): Employed a radial basis function (RBF) kernel; the cost parameter was tuned via cross-validation.

Gradient Boosting Machine (GBM): Utilized a Bernoulli distribution; the number of trees, interaction depth, and shrinkage were optimized.

eXtreme Gradient Boosting (XGBoost): Used a binary logistic objective; early stopping was applied during cross-validation to prevent overfitting.

Feed-Forward Neural Network (NN): A single-hidden-layer architecture with 8 neurons (the number of neurons was determined via a grid search during cross-validation on the training set, considering common candidates such as 4, 8, and 16) and weight decay regularization; training used the Adam optimizer.

Stacked Ensemble: A Ridge regression meta-learner was trained using out-of-fold predictions from the above base learners (RF, SVM, GBM, XGBoost, LR) generated during cross-validation on the training set, preventing leakage.

### 2.6 Model evaluation

The final models, trained on the entire training set with optimal hyperparameters, were evaluated only once on the hold-out test set. The area under the receiver operating characteristic curve (AUC) was the primary metric. Secondary metrics included accuracy, sensitivity, specificity, positive predictive value (PPV), negative predictive value (NPV), and the Brier score. The classification threshold was fixed by maximizing Youden’s index on the training set’s cross-validated ROC curve. The DeLong test was used to compare AUCs between models on the test set, with *p*-values adjusted for multiple comparisons using the Holm method. Model calibration was assessed on the test set using calibration plots, the calibration slope and intercept, and the Hosmer-Lemeshow test. Decision Curve Analysis (DCA) was performed to evaluate clinical utility across a range of threshold probabilities.

### 2.7 Exploratory analysis of atrophy severity

This analysis was conducted on the subset of CAG patients within the training set for methodological consistency. Patients with mild versus moderate atrophy were compared using appropriate statistical tests (independent samples t-test, Wilcoxon rank-sum test, Chi-square test, or Fisher’s exact test) to explore factors associated with severity. A two-sided *p*-value < 0.05 was considered statistically significant.

### 2.8 Statistical software

All analyses were performed using R software (version 4.5.0). Key R packages included caret (version 6.0–94) for data splitting and model training, pROC (version 1.18.5) for ROC analysis, randomForest (version 4.7–1.1), xgboost (version 1.7.7.1), and rmda (version 1.6) for Decision Curve Analysis. A fixed random seed was set prior to the initial data split to ensure complete reproducibility of all results.

## 3 Results

### 3.1 Baseline characteristics of the entire cohort

A total of 222 subjects were included in this study. Following the stratified random split, the training set comprised 178 subjects and the independent test set 44 subjects. The demographic and serum biomarker profiles of the entire cohort, stratified by the pathological diagnosis into chronic atrophic gastritis (CAG, *n* = 68) and non-atrophic gastritis (NAG, *n* = 154) groups, are presented in Table 1.

**Table 1:** Baseline characteristics of the study population.

Variable	Overall (*n* = 222)	NAG (*n* = 154)	CAG (*n* = 68)
Age, years	54.56 ± 12.48	52.80 ± 12.50	58.56 ± 10.80
Male, *n* (%)	143 (64.41%)	97	46
PGI, μg/L	74.90 ± 58.74	82.45 ± 41.23	57.81 ± 31.45
PGII, μg/L	13.67 ± 12.27	12.28 ± 7.54	16.81 ± 9.67
PGI/PGII ratio	6.87 ± 3.38	7.76 ± 3.89	4.85 ± 2.51
G-17, pmol/L	12.27 ± 28.13	12.63 ± 32.91	11.46 ± 11.65
Anti-*H. pylori* antibody positive, *n* (%)	91 (40.99%)	46	45

The univariate analysis comparing CAG and NAG groups was performed on the training set (*n* = 178). Univariate analysis revealed that patients in the CAG group were significantly older than those in the NAG group (58.89 ± 8.42 years vs. 52.95 ± 13.69 years, *p* < 0.001). Regarding gastric functional biomarkers, the CAG group exhibited significantly lower serum levels of PGI (58.25 ± 20.21 μg/L vs. 87.22 ± 33.58 μg/L, *p* < 0.001) and a markedly reduced PGI/PGII ratio (4.87 ± 1.82 vs. 7.85 ± 3.31, *p* < 0.001), while PGII levels were higher (16.02 ± 7.57 μg/L vs. 13.00 ± 6.03 μg/L, *p* = 0.012). No significant difference was observed in G-17 levels between the two groups (*p* = 0.51). For categorical variables, the CAG group had a significantly higher prevalence of anti-*H. pylori* antibody positivity (*p* < 0.001), whereas gender distribution was comparable (*p* = 0.351).

### 3.2 Feature selection via multivariate logistic regression on the training set

As detailed in the Methods, all subsequent modeling steps were performed after an initial stratified split. Feature selection was conducted exclusively on the training set (*n* = 178). Variables significant in the univariate analysis of the training set were incorporated into a multivariate logistic regression model (Table 2). The results identified increased age, decreased PGI, a lower PGI/PGII ratio, and positive anti-*H. pylori* antibody status as independent predictors for CAG within the training cohort. PGII lost its significance after adjustment (*p* = 0.822). Multicollinearity diagnostics indicated no substantial multicollinearity. Therefore, the subsequent machine learning models were developed and evaluated using this locked set of four predictors (PGI, PGI/PGII ratio, age, and anti-*H. pylori* antibody status).

**Table 2:** Multivariable logistic regression analysis for predicting CAG, performed on the training set (*n* = 178).

Variable	Odds ratio (OR)	95% confidence interval (CI)	*p*-value
PGI/PGII ratio	0.82	0.68–0.99	0.042*
Age (years)	1.04	1.00–1.09	0.041*
Anti-*H. pylori* antibody (positive)	4.20	1.82–9.71	<0.001***
PGI (μg/L)	0.98	0.97–1.00	0.022*
PGII (μg/L)	1.00	0.96–1.04	0.822

CAG, chronic atrophic gastritis.

*p < 0.05, ***p < 0.001. The variable PGII was not statistically significant (*p* = 0.822) in the multivariable model and was therefore excluded from the subsequent machine learning models. The final locked set of predictors consisted of PGI/PGII ratio, age, Anti-*H. pylori* antibody status, and PGI.

### 3.3 Comparative performance of machine learning models on the independent test set

**Figure 1:**
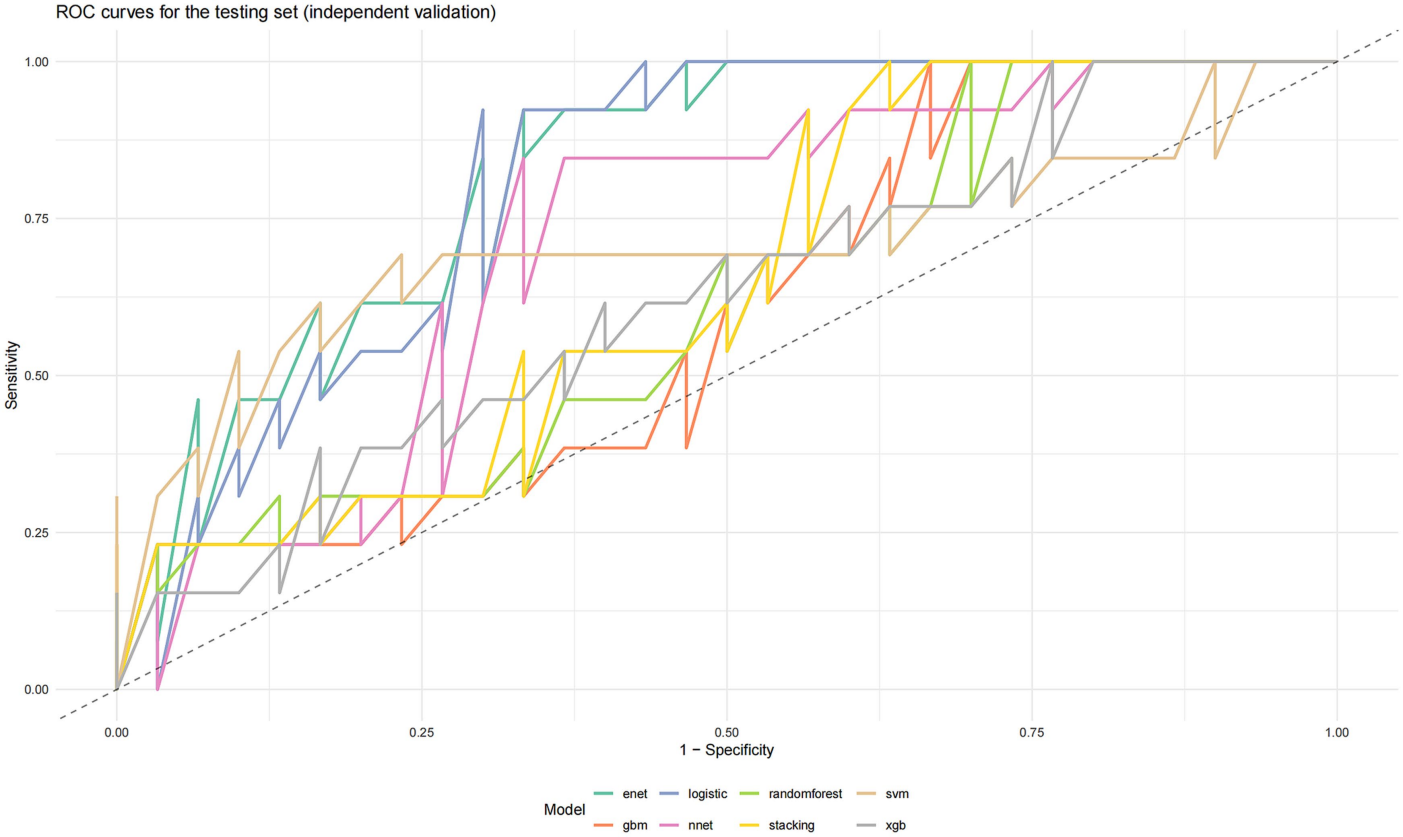
ROC curves for the testing set (independent validation). Models: Elastic Net (enet, testing set AUC = 0.8231), Logistic Regression (logistic, testing set AUC = 0.8103), Random Forest (randomforest, testing set AUC = 0.6128), SVM (svm, testing set AUC = 0.7051), GBM (gbm, testing set AUC = 0.6077), XGBoost (xgb, testing set AUC = 0.6256), Neural Network (nnet, testing set AUC = 0.7154), Stacking Ensemble (stacking, testing set AUC = 0.6513).

Eight models were evaluated on the independent test set (*n* = 44, containing 13 CAG cases). Their discriminative performance is summarized in Table 3 and Figure 1.

**Table 3:** Discriminative performance of models on the independent test set.

Model	AUC (95% CI)	AUPRC (95% CI)
Elastic Net (ENET)	0.823 (0.699–0.947)	0.597 (0.404–0.795)
Logistic Regression (LR)	0.810 (0.683–0.937)	0.604 (0.411–0.797)
Neural Network (NN)	0.715 (0.554–0.876)	0.551 (0.353–0.763)
Support Vector Machine (SVM)	0.705 (0.501–0.909)	0.570 (0.358–0.774)
Stacked Ensemble	0.651 (0.478–0.825)	0.565 (0.356–0.760)
XGBoost (XGB)	0.626 (0.442–0.810)	0.556 (0.356–0.786)
Random Forest (RF)	0.613 (0.429–0.796)	0.587 (0.375–0.790)
Gradient Boosting Machine (GBM)	0.608 (0.426–0.789)	0.567 (0.365–0.767)

The key findings are as follows:

Superior and Robust Performance of Simple Models: The Elastic Net (ENET) and Logistic Regression (LR) models achieved the highest test set AUCs (0.823 and 0.810, respectively) and competitive AUPRCs. Their performance was highly consistent between training and testing phases, indicating minimal overfitting and excellent generalizability.

**Figure 2:**
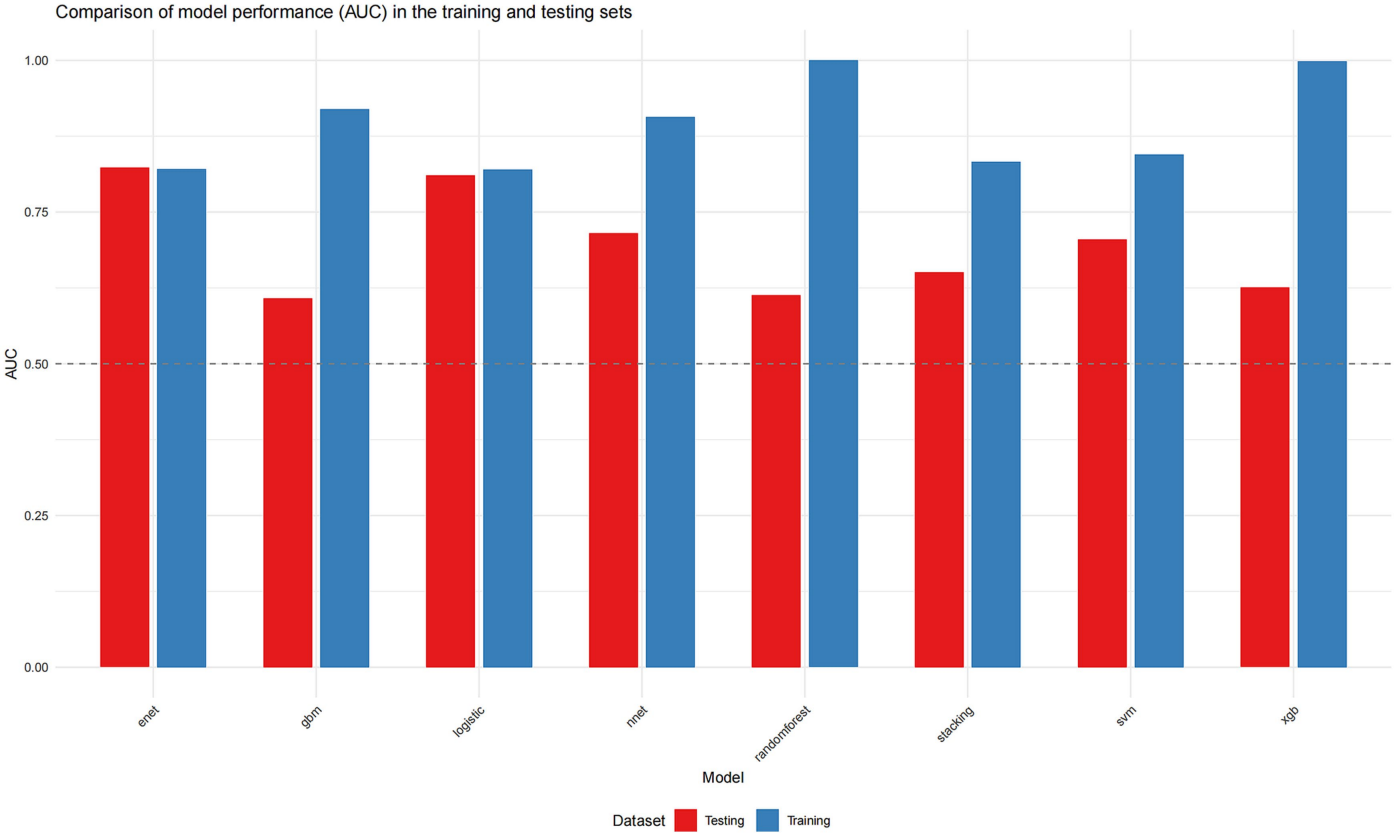
Comparison of model performance (AUC) in the training and testing sets. Logistic regression (training AUC = 0.8201, testing AUC = 0.8103), random forest (training AUC = 0.9999, testing AUC = 0.6128), SVM (training AUC = 0.8450, testing AUC = 0.7051), GBM (training AUC = 0.9196, testing AUC = 0.6077), XGBoost (training AUC = 0.9984, testing AUC = 0.6256), neural network (training AUC = 0.9069, testing AUC = 0.7154), elastic net (training AUC = 0.8205, testing AUC = 0.8231), stacking ensemble (training AUC = 0.8330, testing AUC = 0.6513).

Overfitting in Complex Models: In contrast, tree-based ensemble models (Random Forest, GBM, XGBoost) showed severe overfitting, evidenced by near-perfect training AUCs (0.998–1.000) but substantially lower test AUCs (0.608–0.626). The Neural Network (NN) and Support Vector Machine (SVM) models also demonstrated a marked drop in performance from training to test set (Figure 2).

**Figure 3:**
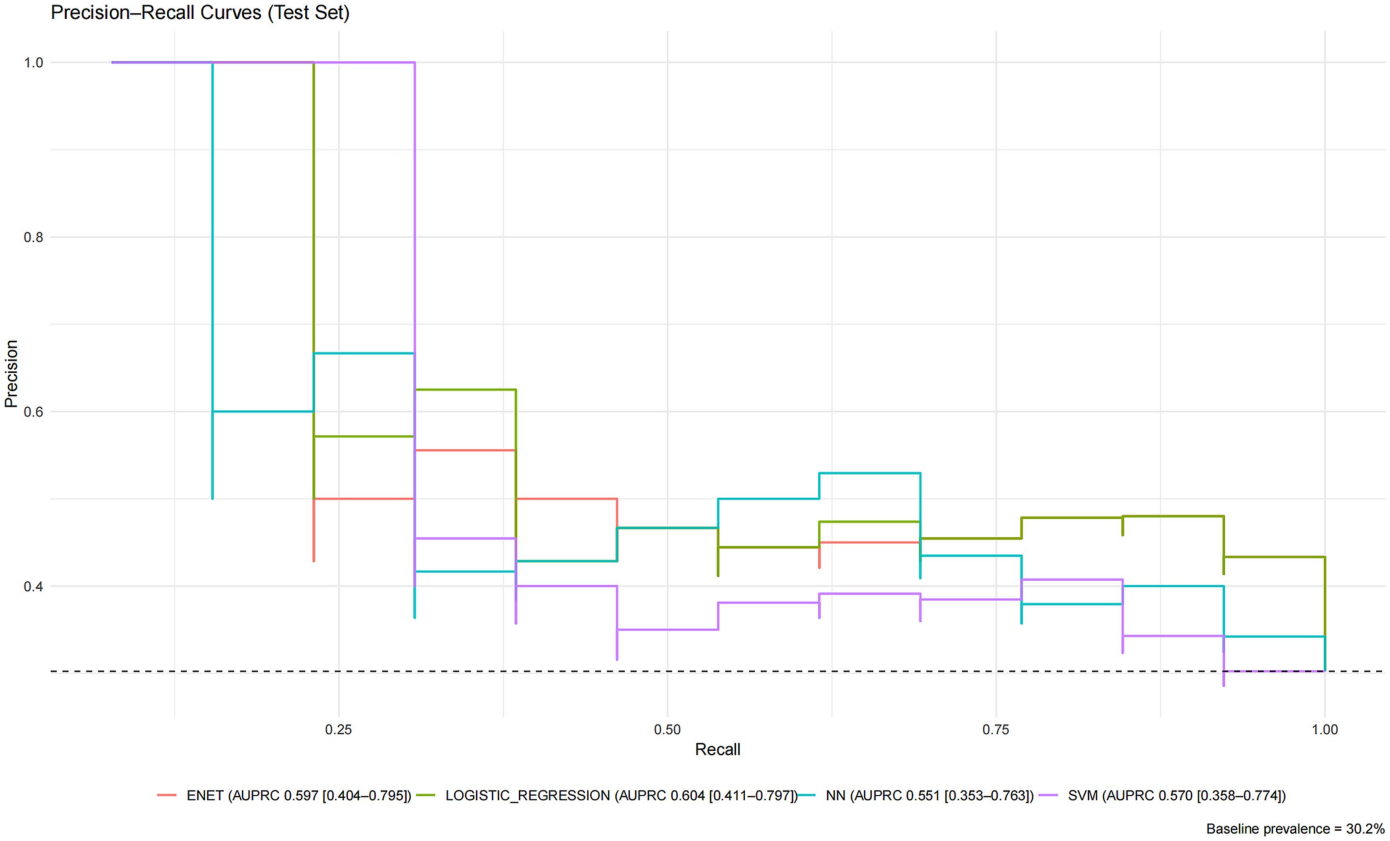
Precision-recall curves. PR curves are shown for the neural network (NN), support vector machine (SVM), logistic regression (LR), and elastic net (ENET). The legend reports AUPRC with 95% CIs: NN 0.551 [0.353–0.763], SVM 0.570 [0.358–0.774], logistic regression 0.604 [0.411–0.797], and ENET 0.597 [0.404–0.795]. The dashed horizontal line marks the baseline prevalence 30.2%. AUPRC was computed as average precision (AP); 95% CIs were obtained via stratified bootstrap (*B* = 2,000).

Statistical Comparisons and Uncertainty: Pairwise DeLong tests revealed that the AUCs of ENET and LR were statistically significantly higher than those of the overfitted tree-based models and the stacked ensemble (e.g., ENET vs. RF, *p* = 0.0021; LR vs. GBM, *p* = 0.0022). However, the AUC differences between ENET/LR and the non-overfitted complex models (SVM, NN) were not statistically significant (e.g., ENET vs. SVM, *p* = 0.142; LR vs. NN, *p* = 0.092). The Precision-Recall (PR) curves for the top four models (ranked by test set AUC) are presented in Figure 3. These models also exhibited wide confidence intervals for AUPRC (Table 3), reflecting the considerable uncertainty inherent in evaluating models with a small test set containing only 13 events.

### 3.4 Head-to-head comparison and clinical utility of the top models

Given their robust performance, the Elastic Net (ENET) and Logistic Regression (LR) models were compared in detail at their optimal thresholds, which were determined by maximizing Youden’s index on the training set. Their classification performance on the independent test set is summarized in Table 4.

**Table 4:** Detailed performance comparison of the top two models at the optimal threshold.

Metric	ENET	LR
Threshold	0.208	0.206
Sensitivity	0.923	0.923
Specificity	0.667	0.700
PPV	0.545	0.571
NPV	0.952	0.955
Accuracy	0.744	0.767
Youden’s Index	0.590	0.623

Both models demonstrated excellently high sensitivity (0.923) and negative predictive value (NPV > 0.95), underscoring their strength as tools for “ruling out” CAG. The LR model showed marginally better performance in specificity, positive predictive value (PPV), overall accuracy, and Youden’s index.

**Figure 4:**
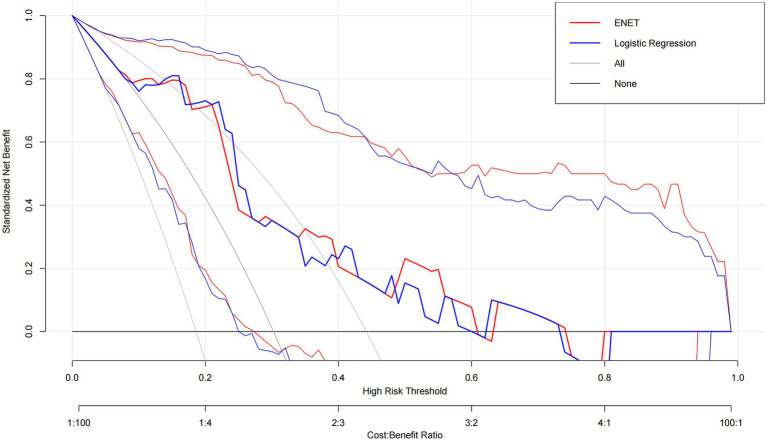
Decision Curve Analysis. Standardized net benefit curves for the elastic net (ENET) and logistic regression (LR) across threshold probabilities 0.01–0.99, compared with the “treat-all” and “treat-none” strategies. The event prevalence is 30.2%. Both models achieve sustained positive net benefit exceeding “treat-all” over the following threshold ranges: ENET: 0.08–0.60 (and 0.64–0.74); LR: 0.09–0.60 (and 0.63–0.73). Shaded ribbons indicate 95% confidence intervals.

Decision Curve Analysis (DCA) further quantified their potential clinical utility across a range of threshold probabilities (Figure 4). In the test set (CAG prevalence = 30.2%), both ENET and LR provided a superior net benefit compared to the default “treat-all” strategy across nearly identical, clinically relevant threshold ranges (ENET: 0.08–0.60 & 0.64–0.74; LR: 0.09–0.60 & 0.63–0.73), with their net benefit curves being nearly indistinguishable.

**Figure 5:**
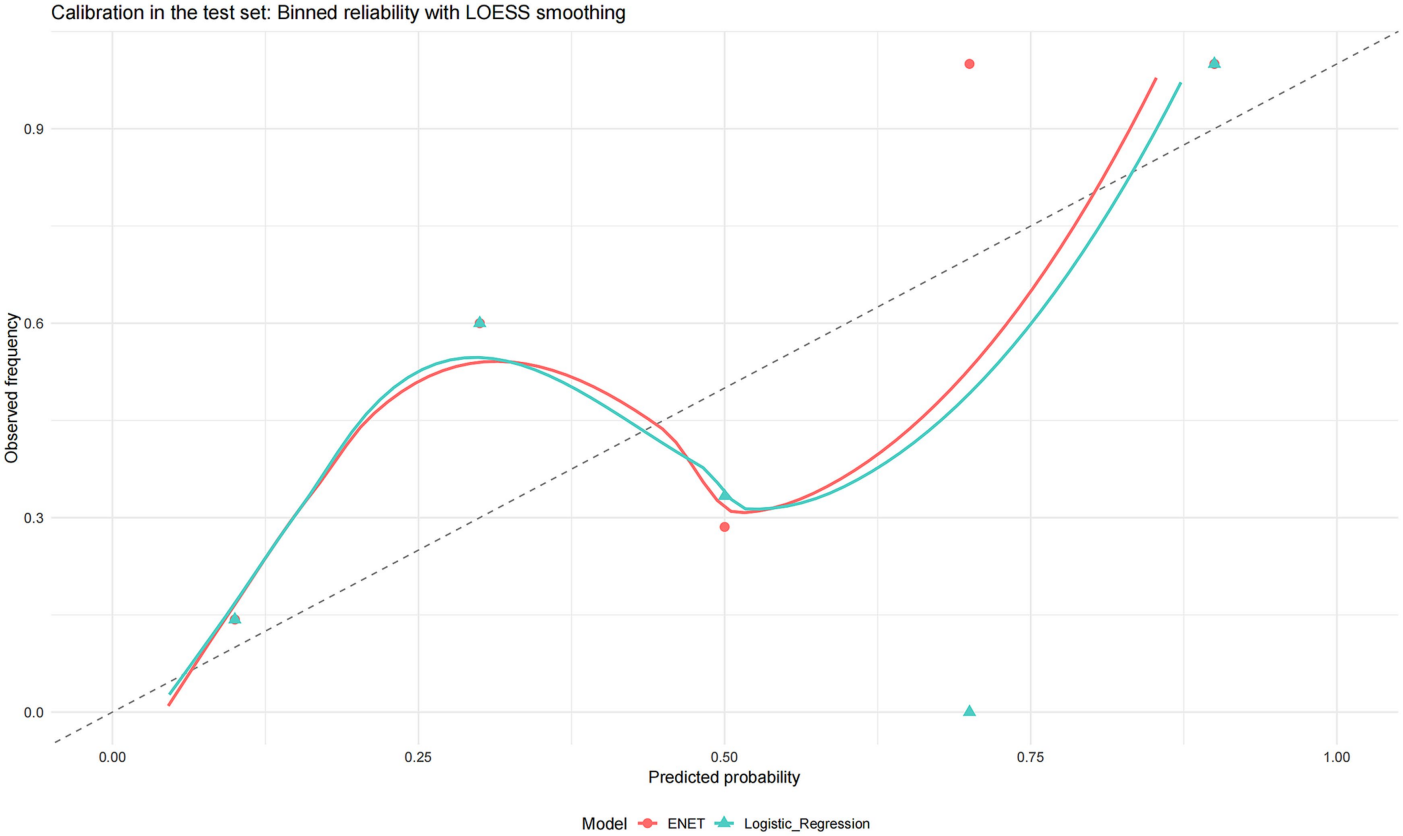
Calibration in the test set binned reliability with LOESS smoothing. Calibration plots for the two shortlisted models (elastic net and logistic regression). Points show binned observed event frequencies against the mean predicted probabilities (approximately equal-sized bins; point size reflects bin size), while solid curves are LOESS smoothers of observed vs. predicted risk. The dashed 45° line denotes perfect calibration.

Finally, both models demonstrated acceptable calibration in the test set (Figure 5).

### 3.5 Model interpretation

**Figure 6:**
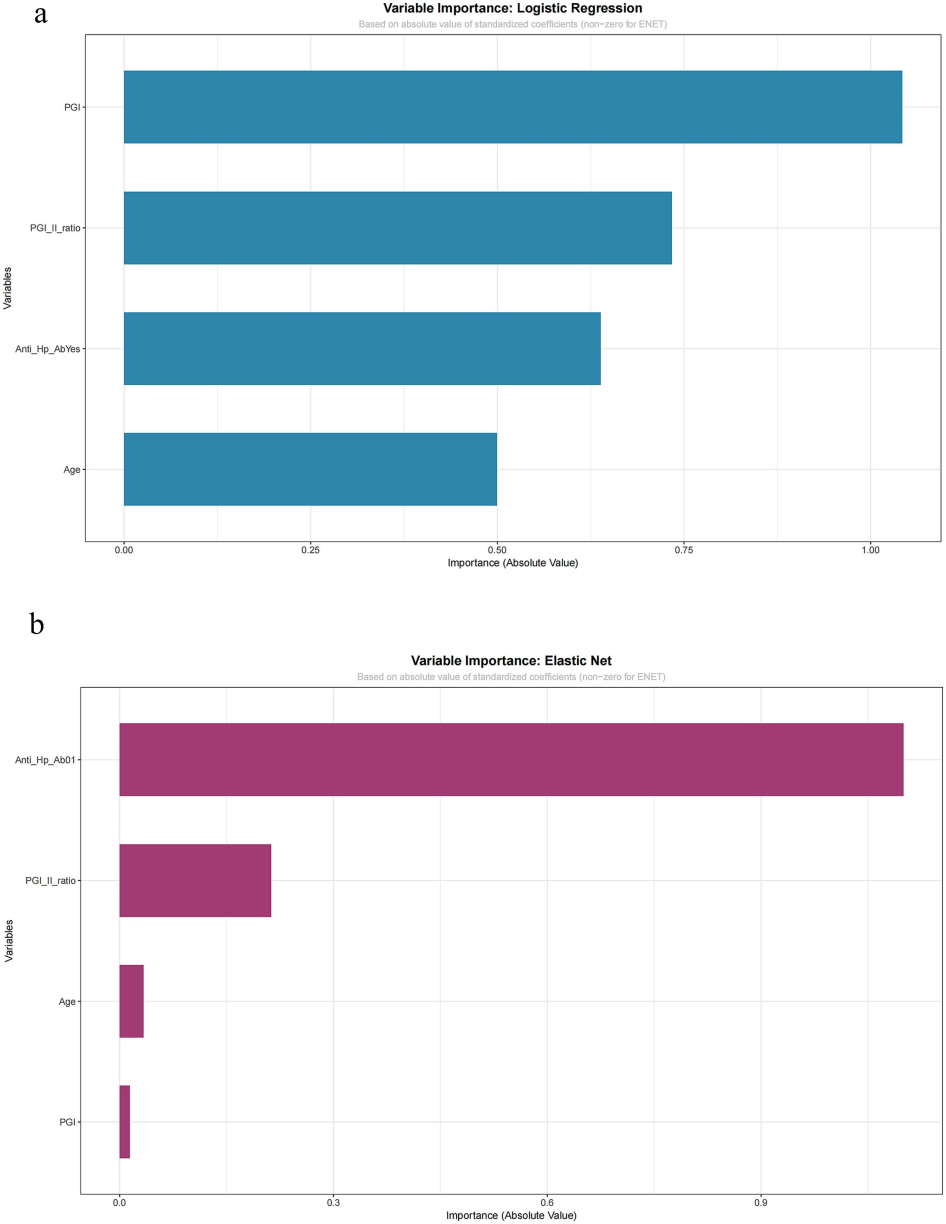
Variable importance: logistic regression and elastic net. Importance is quantified by the absolute value of standardized coefficients. **(a)** Logistic regression model. **(b)** Elastic net model. Variables shown include pepsinogen I (PGI), pepsinogen I/II ratio, anti-HP-Ab, and age.

Given their optimal and robust performance, the Logistic Regression and Elastic Net models were examined for interpretability. The ranking of variable importance, based on the absolute value of standardized coefficients, differed between the two methods (Figure 6a for Logistic Regression, Figure 6b for Elastic Net). In Logistic Regression, PGI had the highest importance value, followed by the PGI/PGII ratio and anti-*H. pylori* antibody status. In Elastic Net, anti-*H. pylori* antibody status was ranked highest, followed by the PGI/PGII ratio, with PGI having the lowest relative importance.

Critically, despite differences in ranking, both models agreed on the direction of association for key predictors, as evidenced by their raw coefficients: anti-*H. pylori* antibody positivity was a consistent positive predictor, while a higher PGI/PGII ratio was a consistent negative (protective) predictor of CAG. The variation in the absolute importance ranking of PGI highlights the sensitivity of this metric to different modeling techniques, particularly in smaller datasets. This finding underscores that while both interpretable models confirm the central, directionally consistent role of serological and infectious biomarkers, the precise quantification of their relative influence should be viewed as exploratory.

### 3.6 Exploratory analysis of atrophy severity in the training subset

In the subgroup of patients with pathologically confirmed CAG (*n* = 68), we compared those with mild (*n* = 53) and moderate (*n* = 15) atrophy (Table 5). Fisher’s exact test indicated a significant association between gender and atrophy severity (*p* = 0.026). However, no significant differences were observed between the mild and moderate groups regarding age, PGI, PGII, the PGI/PGII ratio, G-17 levels, or *H. pylori* infection status (all *p* > 0.05).

**Table 5:** Univariate comparison between mild and moderate atrophic gastritis patients.

Variable	Test	Test statistic	*p* value
Age (years)	Independent-samples *t* test	−0.65	0.520
PGI (μg/L)	Independent-samples *t* test	0.91	0.371
PGII (μg/L)	Independent-samples *t* test	−0.81	0.430
PGI/PGII ratio	Independent-samples *t* test	0.41	0.684
G-17 (pmol/L)	Independent-samples *t* test	0.90	0.378
Sex	Fisher’s exact test	–	0.026
Anti-*H. pylori* antibody	Chi-square test	0.78	0.378

## 4 Discussion

This study developed and rigorously validated multiple predictive models for chronic atrophic gastritis (CAG) using readily accessible clinical parameters. Our key finding is that simple, interpretable linear models—Elastic Net (ENET) and Logistic Regression (LR)—demonstrated the most robust and generalizable performance on an independent test set, achieving the highest area under the curve (AUCs of 0.823 and 0.810, respectively) with excellent sensitivity (0.923) and negative predictive value (NPV > 0.95) (11–13). In contrast, more complex machine learning (ML) algorithms, particularly tree-based ensembles, exhibited significant overfitting, underscoring that model complexity does not inherently guarantee superior performance in this context with a moderate sample size and a limited set of biologically plausible predictors (14–17).

A pivotal observation from our model comparison is the distinct pattern of performance consistency. While some complex models (e.g., SVM) achieved test-set AUCs numerically comparable to the linear models, tree-based ensembles (Random Forest, GBM, XGBoost) showed a dramatic decline from near-perfect training performance to substantially lower test performance, indicating overfitting. It is crucial to acknowledge that the limited number of CAG events (*n* = 13) in our independent test set results in wide confidence intervals for performance metrics like AUC. Therefore, small numerical differences between the top-performing models should be interpreted with caution, and the core finding rests on the observed contrast in robustness between model families, rather than on precise point estimates.

Our study design involved an important methodological choice: feature selection was performed using multivariate logistic regression on the training set. This approach prioritizes parsimony and interpretability and helps mitigate overfitting. However, it also constitutes a potential trade-off, as it may constrain the ability of subsequent non-linear ML algorithms to discover complex interactions beyond the selected linear combination of predictors. Thus, our comparison primarily evaluates the performance of different algorithms when applied to a curated set of strong, clinically coherent predictors.

The superior and stable performance of ENET and LR models carries significant clinical implications. Their exceptionally high sensitivity and NPV suggest a primary utility in “ruling out” CAG in a pre-endoscopic triage setting for patients already under clinical suspicion. Decision Curve Analysis confirmed that both ENET and LR provided a superior net benefit compared to a “treat-all” strategy across clinically relevant threshold probabilities, reinforcing their potential practical value for risk stratification in secondary care (18–20). It is important to note, however, that our cohort consisted of patients who underwent endoscopy, indicating a clinically selected population. Therefore, this “rule-out” performance must be validated in true screening cohorts of asymptomatic individuals before widespread application, as performance may differ in a lower-prevalence, unselected population.

The predictors retained in our final models—age, PGI, PGI/PGII ratio, and *H. pylori* antibody status—are firmly grounded in the pathophysiology of CAG. Multivariate analysis confirmed them as independent risk factors, aligning with established knowledge: advancing age and *H. pylori* infection are key drivers of chronic gastric inflammation and atrophy, while decreased PGI and a reduced PGI/PGII ratio directly reflect the loss of functional gastric chief cells (21–23). Notably, PGII did not retain independent significance, reinforcing the superior value of the PGI/PGII ratio as a composite marker of mucosal status. The lack of association with G-17 in our study may relate to the specific topography of atrophy in our cohort or the interplay between acid secretion and G-17 dynamics, warranting investigation in studies with detailed anatomic mapping.

A pivotal observation from our model comparison is that the performance gains from complex non-linear ML models over traditional linear methods were minimal and statistically non-significant. While models like SVM and NN achieved numerically comparable test-set AUCs, DeLong tests confirmed no statistically significant difference between them and the top-performing linear models (ENET/LR). More critically, ensemble tree models displayed severe overfitting. This pattern strongly suggests that the relationships between our four selected predictors and CAG are predominantly linear or monotonic. With a limited number of strong, clinically coherent predictors, a simple linear model adequately captures the underlying signal, whereas complex models risk fitting noise in the training data, compromising generalizability. This finding echoes the growing consensus that for many clinical prediction problems with structured data, well-specified regression models can be as effective as, and more stable than, complex black-box algorithms, especially in smaller datasets.

Our interpretation of variable importance, while directionally consistent across ENET and LR, yielded different rankings of absolute importance. This discrepancy highlights the sensitivity of such metrics to modeling techniques and sample size, suggesting that while the identified predictors are core to CAG risk, their precise quantified relative contributions should be viewed as exploratory.

This study has several limitations that must be acknowledged. First, the modest sample size, particularly the small number of CAG events (*n* = 13) in the independent test set, leads to considerable uncertainty in performance estimates, as evidenced by the wide confidence intervals. This statistical imprecision limits strong conclusions regarding subtle differences between models. Second, our binary outcome (CAG vs. non-CAG) lacks the granularity of established staging systems like OLGA/OLGIM. Consequently, our model is designed to detect the presence of any atrophy, not to discriminate between mild and advanced atrophy stages or to predict specific cancer risk, which is a crucial next step for refined clinical decision-making. Third, the single-center, retrospective design with an endoscopy-indicated cohort introduces spectrum bias, limiting the immediate generalizability of our findings to asymptomatic screening populations and underscoring the necessity for external validation in such settings. Finally, as noted, the pre-selection of features may have limited the exploration of more complex predictive patterns (24, 25).

In conclusion, this study demonstrates that a parsimonious model based on age, PGI, the PGI/PGII ratio, and *H. pylori* serology can effectively serve as a triage tool for identifying individuals at risk for CAG within a clinical population referred for endoscopy evaluation. The Elastic Net and Logistic Regression models emerged as the most robust and clinically interpretable options. Our comparative analysis highlights that in this predictive context, increased model complexity did not translate to better generalization and was, in some cases, compromised by overfitting. Future research should focus on: (1) external validation of these models in larger, multi-center prospective cohorts encompassing both symptomatic and asymptomatic individuals; (2) integration of the model with detailed pathological staging (OLGA/OLGIM) to predict high-risk lesions; and (3) conducting impact studies to evaluate their effect on endoscopic resource utilization and patient outcomes before clinical implementation can be considered.

## Data Availability

The raw data supporting the conclusions of this article will be made available by the authors without undue reservation.
